# Bento Box: An Interactive and Zoomable Small Multiples Technique for Visualizing 4D Simulation Ensembles in Virtual Reality

**DOI:** 10.3389/frobt.2019.00061

**Published:** 2019-07-23

**Authors:** Seth Johnson, Daniel Orban, Hakizumwami Birali Runesha, Lingyu Meng, Bethany Juhnke, Arthur Erdman, Francesca Samsel, Daniel F. Keefe

**Affiliations:** ^1^Interactive Visualization Lab, Department of Computer Science, University of Minnesota, Minneapolis, MN, United States; ^2^Research Computing Center, University of Chicago, Chicago, IL, United States; ^3^Department of Mechanical Engineering, Earl E. Bakken Medical Devices Center, University of Minnesota, Minneapolis, MN, United States; ^4^Texas Advanced Computing Center, University of Texas, Austin, TX, United States

**Keywords:** virtual reality, ensemble visualization, comparative visualization, 3D user interfaces, small multiples

## Abstract

We present Bento Box, a virtual reality data visualization technique and bimanual 3D user interface for exploratory analysis of 4D data ensembles. Bento Box helps scientists and engineers make detailed comparative judgments about multiple time-varying data instances that make up a data ensemble (e.g., a group of 10 parameterized simulation runs). The approach is to present an organized set of complementary volume visualizations juxtaposed in a grid arrangement, where each column visualizes a single data instance and each row provides a new view of the volume from a different perspective and/or scale. A novel bimanual interface enables users to select a sub-volume of interest to create a new row on-the-fly, scrub through time, and quickly navigate through the resulting virtual “bento box.” The technique is evaluated through a real-world case study, supporting a team of medical device engineers and computational scientists using *in-silico* testing (supercomputer simulations) to redesign cardiac leads. The engineers confirmed hypotheses and developed new insights using a Bento Box visualization. An evaluation of the technical performance demonstrates that the proposed combination of data sampling strategies and clipped volume rendering is successful in displaying a juxtaposed visualization of fluid-structure-interaction simulation data (39 GB of raw data) at interactive VR frame rates.

## 1. Introduction

Science and engineering workflows increasingly rely upon ensembles—“concrete distributions of data, in which each outcome can be uniquely associated with a specific run or set of simulation parameters” (Obermaier and Joy, 2014). Analyzing these ensembles is a challenging task that involves not just understanding specific data values and trends but also making comparisons. Visualization can help, and recent ensemble visualization research has made it possible to: (1) manage and render some of the large datasets that are encountered with ensembles (Vohl et al., 2016); (2) use interactive techniques to navigate through large ensemble parameter spaces (Sedlmair et al., 2014), including using both local-to-global (Coffey et al., 2013) and global-to-local approaches (Bruckner and Moller, 2010); and (3) use simulation steering to explore “what if” scenarios (Waser et al., 2010, 2014). Unfortunately for scientists and engineers, much work remains—successful ensemble visualization requires not just a minor adjustment of the traditional visualization pipeline but rather a significant reworking. Major current problems include:

- The lack of connection between the research on ensemble visualization and theoretical research on comparative visualization (Gleicher et al., 2011; Gleicher, 2017; Kim et al., 2017) which discusses fundamental trade-offs between perceptual strategies required for making comparisons, such as *juxtaposition (side-by-side), superposition (overlayed), interchangeable (animating through or interactively switching between viewing a single data instance at a time), explicit encoding (e.g., computing the difference between data instances), and hybrid approaches*.
- The lack of ensemble visualization techniques, including user interfaces, designed specifically for use in virtual reality (VR) environments. We know perspective-tracked, stereoscopic displays can outperform desktop tools for spatial perception tasks (Ware and Mitchell, 2005, 2008), but designing effective VR visualization tools is challenging and requires synthesizing and refining user interface research results on bimanual interaction (Hinckley et al., 1997), navigation (Stoakley et al., 1995), selection (Bowman and Hodges, 1997), and manipulation (Mapes and Moshell, 1995) (citations limited here to some early, seminal works); these VR and 3D user interface research results are not always widely cited and used in scientific visualization.
- The need for additional examples (i.e., case studies) of how to organize data and perform rendering of different types of ensembles. This is important because rendering at the ensemble scale requires fundamentally different approaches for 4D fluid dynamics computed on unstructured grids (explored here) as compared to, for example, 2D maps and imagery (Javed et al., 2012). In addition, visualizing spatial relationships and interaction between data parts in multimodel scenarios (i.e., fluid and structure interactions) is rarely explored (Kehrer and Hauser, 2013).

This paper addresses the specific unsolved challenge of visualizing moderate-sized ensembles (e.g., containing on the order of 10 data instances) of state-of-the-art, time-varying fluid-structure interaction simulations run on high-performance computing platforms. This size of ensemble is useful to study because it is large enough to present challenges in rendering and visual comparison but not so large as to rule out the possibility of visualizing the entire ensemble simultaneously. The strategies developed for this scale can likely be combined with others (e.g., filtering) to address larger ensembles.

This paper also focuses on VR-based visualization. The rationale for VR is based on the data. With interdisciplinary collaborators, we are studying simulations of blood flow through the heart, and this requires analyzing complex spatial relationships, such as subtle differences in 4D vortical structures. Formally, low-level perceptual studies suggest that perspective-tracked, stereoscopic visualization can facilitate understanding complex spatial relationships found in 3D data (Ware and Mitchell, 2005, 2008), providing evidence for the likely utility of VR in our work. Informally, our collaborators have consistently cited an improved ability to see spatial patterns in the data with VR and repeatedly demanded to analyze the data using VR over the course of a 5+ year project. Taken together with the fact that there is no longer a financial barrier to using VR for scientific visualization, we take this as strong motivation.

Our proposed solution builds upon recent theory on comparative visualization in 2D contexts (Gleicher et al., 2011; Gleicher, 2017) as well as 3D and 4D (3D + time) contexts (Kim et al., 2017). Specifically, we adopt the fundamental approach to visual comparison known as *juxtaposition* and adapt it to suit VR-based volumetric visualization.

The rationale for the juxtaposition strategy as compared to the *interchangeable* strategy (another fundamental approach discussed in the literature) can be summarized by the visualization rule of thumb, “eyes beat memory” (Munzner, 2014); making comparisons is easier if we can see the items to compare simultaneously rather than trying to remember one or more previously viewed items. The rationale for juxtaposition as compared to *superposition* is specific to the data of interest. These 4D data are so dynamic and the spatial patterns so complex, that we rule out superposition due to the extreme complexity and occlusion issues that would occur when rendering 10 blood flow datasets in the same visual space. The final fundamental approach to comparative visualization discussed in the literature is *explicit encoding*. Explicit encoding is an excellent approach and could be used within an extended version of our tool (future work); however, designing a new explicit encoding for comparison is so dataset specific that it often becomes its own research project with results that may not translate well to other datasets.

This leads us toward a juxtaposition approach, in general, however, juxtaposition is not perfect, and the trade-offs are what makes designing an effective ensemble visualization such a challenge. For example, one concern with juxtaposition is that when visuals to compare are viewed side-by-side, the viewer's eyes must move back and forth between the visuals in order to find correspondences and notice differences. This takes effort and time, and the naïve approach of simply rendering each data instance, one next to the other, is unlikely to be the most useful, especially when the key differences are subtle and appear in small sub-regions of the volume data. Our strategy to mitigate this is to make it possible for users to interactively design a spatial layout that places all of the volumetric features of interest as close as possible to each other. We call the resulting tool, which neatly slices and places data into an organized grid of sub-volumes, *Bento Box*.

**Figure 1:**
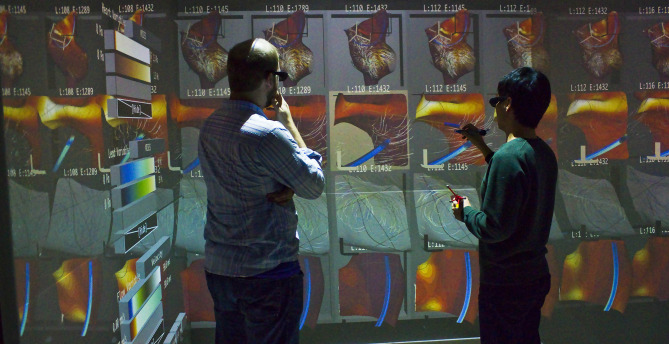
Bento Box is a virtual reality visualization and 3D user interface technique for comparative analysis of data ensembles, such as this set of 10 time-varying simulations of blood flow around a cardiac lead in the right atrium of the heart. Each *column* shows a simulation with different parameters, here varying the length and stiffness of the lead. Each *row* shows a different view of the data. The top row is a zoomed-out overview. Users add additional rows of complementary, zoomed-in views during analysis.

Figure 1 shows Bento Box in use with the medical device design application. Diving into this example just a bit now, we know engineers need to analyze several important sub-volumes of data within the right atrium of the heart, including: (1) the stress in the right atrial appendix, (2) the speed of the flow in the main vortex that forms, and (3) the stress through a cross-section of the lead inserted in the heart. Engineers must analyze all of these aspects and more, making comparisons across each instance, in order to completely understand the ensemble. A key design goal is, therefore, to make it as easy as possible for users to compare a specific volumetric feature (we will call this a sub-volume of interest) while also switching focus easily back and forth between several sub-volumes of interest. The Bento Box technique accomplishes just this.

Bento Box naturally draws upon and advances research that is relevant to both the VR and Visualization research communities, and we expect this paper to be especially relevant to the growing number of researchers that span the two communities. Our contributions necessarily span both technical areas and include:

- The design of Bento Box, a VR visualization technique for comparative analysis of 4D data ensembles.
- A novel bimanual 3D user interface that supports: (1) zooming and reframing the Bento Box, (2) selecting sub-volumes of interest, (3) navigating within these sub-volumes, and (4) specifying multiple critical times to compare.
- A case study that describes how to apply rendering and data-sampling algorithms for visualizing Fluid-Structure-Interaction (FSI) simulation data for the first time to multiple instances of these data simultaneously in stereoscopic, head-tracked VR.
- A performance evaluation of the rendering and data sampling strategies applied to the cardiac application.
- User feedback from the interdisciplinary application.

The paper is organized as follows. After additional discussion of related work, we present the Bento Box technique. The key novel aspects here are the visual layout and VR user interface, both of which are likely to generalize to use with other volumetric datasets. Then, we present the application developed as part of a multi-year interdisciplinary team-science research project; this includes important details, such as data sampling strategies, that are required to make the technique practical for use with modern, actively researched 4D flow datasets. The real-world application serves as an initial evaluation of the technique, and both user feedback and rendering performance are reported. Finally, we discuss limitations and future work as well as conclusions.

## 2. Related Work

Several areas of related work are relevant.

### 2.1. Ensemble Visualization and Comparative Visualization

Sedlmair et al. present a conceptual framework that is useful for characterizing ensemble visualizations, including several fundamental approaches for navigating through a parameter space (Sedlmair et al., 2014). One such approach is “local-to-global,” as demonstrated in Design by Dragging (Coffey et al., 2013). Here, for the most part, the user focuses on visualizing just a single instance of data at a time, and the emphasis within the user interface is on making it easy to transition from the current instance to others that make up the ensemble. Connecting to the literature on comparative visualization, this work uses what Kim et al. describe as an “interchangeable approach” augmented with animated transitions (Kim et al., 2017). Recall, this approach has a key theoretical perceptual limitation—the data to compare are not simultaneously visible.

Bruckner and Möller present an alternative (Bruckner and Moller, 2010), characterized by Sedlmair et al. as “global-to-local.” Here, the visualization system starts with an overview in the form of thumbnail images. The thumbnails enable juxtaposed comparative visualization at the overview level. Interactive filters then make it possible for users to narrow the search space to the most promising subset of the ensemble for closer inspection. Individual data instances can then be examined one at a time in a single 3D view window. In comparing with Bento Box, a major strength of this approach is the filtering, which enables the technique to scale to larger ensembles. However, a drawback relative to Bento Box is that detailed analyses of the final volume data are done on the desktop with a single 3D window at a time. Comparing this as well as related ensemble visualization tools that include some form of juxtaposed comparison, for example World Lines and follow-on systems (Waser et al., 2010, 2014), a key difference with Bento Box is supporting not just comparison via overview thumbnails in the top row of the widget, but also detailed comparisons of even subtle variations across multiple volumes that can all be viewed simultaneously from multiple vantage points in VR.

Vohl et al. (2016) have done some of the most impressive work in ensemble visualization from a systems perspective, multiple data instances may be displayed juxtaposed for collaborative analysis using an ecosystem of displays (desktop, mobile, and VR); in VR/large-screen mode the system uses a handheld tablet as a display controller. In contrast, Bento Box is optimized for VR and introduces a bimanual user interface for not just rotating data and assigning data instances to specific sub-displays but also for selecting sub-volumes of interest in 3D space and reframing the virtual display for comfortable viewing. This is intentionally designed to enable a fluid style of data exploration so users do need to look away from the data when operating the interface.

Chi et al. (1997) introduced a spreadsheet-inspired layout for data visualization, highlighting that “Custom Tabular Layouts Enable Comparisons” and including data processing operators, such as subtracting one data instance from another. Working with volumetric data where explicit “difference” encodings are often more complex to compute, Jankun-Kelly and Ma (2001) combine volume rendering and a spreadsheet-like interface to visually explore an ensemble. Axes may represent ensemble parameters, time, color maps, or transfer functions. Bento Box extends the concept of a 2D grid, small-multiples-style layout to one where users to interactively set the views along the vertical axis based on spatial croppings of volume data. In addition, Bento Box reinterprets the approach as a native VR visualization technique with integrated 3D user interface.

Finally, Alabi et al. (2012) take an innovative approach to comparative visualization where multiple surface models are sliced and then displayed in an interleaved 3D space. The result is visually distinct, but the approach shares a similar underlying theoretical reasoning with Bento Box. Both techniques prefer juxtaposition to superposition, reasoning that the resulting 3D visual display would be too complex with superposition. However, both also recognize that the spatial separation that comes with a naïve application of juxtaposition (one complete dataset next to another, then another,…) is also problematic. The solution in both involves slicing the data, placing corresponding sub-volumes as close together as possible. The key differences in Bento Box are the use of more complex 4D data, including fluid flow, which would likely not work well with the extreme spatial interleaving Alabi et al. use for surfaces, and the fact that the sliced display can be defined interactively by the user directly within a VR environment. Slicing to pull out subsets of data for comparison has also been used in 2D visualization (e.g., for time-series and image data) (Javed and Elmqvist, 2010; Javed et al., 2012); but, the spatial arrangement and interface are necessarily different when working with volume datasets.

### 2.2. Flow Visualization and Animation

There is a long history of visualizing fluid flows in VR, dating to Bryson's seminal Virtual Wind (Bryson, 1996). Early work in this area relied upon interactive visualization widgets, such as interactively placed particle emitters and streamline rake widgets. This elegantly enables user exploration but it also has a key limitation in that the data are essentially hidden from the display until the user places widgets to reveal them. The Particle Flurries technique takes an opposite approach, using a fleet of carefully seeded particles to present a synoptic animated view of the data (Sobel et al., 2004). For many tasks (e.g., gaining an overall understanding of a flow), we see this as the preferable approach; however, it is not well suited to comparative visualization, as reading 10 animated flow visualizations side-by-side would simply be impossible from a perceptual standpoint. Our approach is a hybrid. Flow visualizations are pre-populated with 3D comets inspired by Mitchell et al.'s carefully designed 2D streaklets (Mitchell et al., 2009), but the default presentation is static rather than animated.

The best role for animation is something that was considered carefully in Bento Box. Prior perceptual research suggests that, although animation can be useful for explaining trends, it is less useful for data analysis tasks (Tversky et al., 2002; Robertson et al., 2008). For identifying trends in the first place, it is often the case that a carefully designed static visualization is better than an animated visualization. In addition, prior VR-based studies suggest that interactive control over time is useful and important to support for some tasks, such as identifying the exact moment of collision by two objects in 3D space (Coffey et al., 2012a). These motivate our approach to default to static views of multiple time steps and support interactive control of time and animated overviews as options. A timeline interface is used; however, the direct manipulation interface introduced by Hentschel et al. (2008), would integrate perfectly into Bento Box and is a planned future addition to the tool.

### 2.3. Bimanual and 3D User Interfaces

Navigation, object selection, and object manipulation are major topics within the VR and 3D user interface research (Bowman et al., 2004). What is unclear from the prior literature is how best to move these techniques from user studies or other application scenarios to complete, high-end visualization applications. For example, there is no prior work that demonstrates how to select sub-volumes of interest within 3D visualizations using the same bimanual controllers that are also used for multi-scale navigation of multiple datasets and for system control (e.g., interacting with menus and timelines).

Bento Box extends the bimanual scene navigation and object manipulation techniques first introduced by Mapes and Moshell (1995), which have since been revisited and revised many times (e.g., Cutler et al., 1997; Keefe et al., 2001). Specifically, Bento Box makes it possible to use these techniques at multiple scales. When the user's hand enters the Bento Box, the technique transitions from a scene-level manipulation to a local object manipulation interface. Another extension is that a laser (ray-casting) based selection is used for easy, coarse selections when working from a distance, but this seamlessly transitions to a cursor, point-based interface for detailed selection of sub-volumes of interest when the hand is held inside the Bento Box. In addition to these smooth interface transitions, Bento Box also includes automated view adjustments for quickly zooming to a subsection of the grid or zooming back out to an overview state.

Building upon early theoretical work in bimanual user interfaces in 2D (Hinckley et al., 1997; Leganchuk et al., 1998) operations are assigned to the hands following the guidance that the non-dominant hand should set the context within which the dominant hand performs, often more precise, operations. Prior work in VR has used these interface design concepts (Keefe et al., 2001, 2007), but in different applications (e.g., 3D painting). There are just a few prior examples of bimanual 3D spatial interfaces for manipulating and navigating through volumetric scientific data (e.g., Coffey et al., 2012b; Laha and Bowman, 2013), and none of these specifically support comparative visualization.

Bento Box also extends research on button overloading in VR (e.g., Zeleznik et al., 2002; Jackson and Keefe, 2016) by providing an example of how this can be usefully employed for data visualization tasks rather than 3D modeling. This is achieved via a state machine that uses the current context defined by the positioning of the hands relative to the body, each other, and/or virtual content to decide how to interpret each button press.

## 3. Bento Box: Concept, Visual Layout, and Interface

This section presents the concept, visual layout, and specific interactive techniques that make up Bento Box. Some figures refer to the cardiac application mentioned earlier as examples, but we defer a detailed description of that application to 4.

We designed Bento Box to run on multiple VR environments that are popular for scientific visualization today, and demonstrate here and in the accompanying video from the Supplementary Material that the current implementation runs on both a high-end 4-wall Cave, which is useful to facilitate discussion with an engineering design group, and a low-cost HTC Vive personal VR display. The minimum required VR hardware is as follows. The technique relies upon a perspective-tracked, stereoscopic VR display and requires input from two 6 degree-of-freedom tracked input devices (i.e., VR wands), one held in each hand. Each device must have two buttons, one primary and one secondary that report separate *button_down* and *button_up* events. These input requirements are a subset of what is available via the hardware for the current-generation HTC Vive. In the 4-wall Cave environment we have used, users hold two tracked 3D pen-like devices (Jackson and Keefe, 2016) to provide the simple button input together with 6-DOF tracking. Our implementation uses the MinVR toolkit (Jackson, 2017) to facilitate deploying the application to these multiple platforms. The technique is designed to be operated by one user, but in practice engineering teams like to stand together and work as a group, looking over an operator's shoulder in the Cave environment pictured in Figure 1.

### 3.1. Concept and Visual Layout

When users enter the environment, they see the “Bento Box” widget, an arranged grid containing multiple views of volume data, floating in front of them as in Figure 1. A 3D cursor is also drawn at the location of each tracked hand.

**Figure 2:**
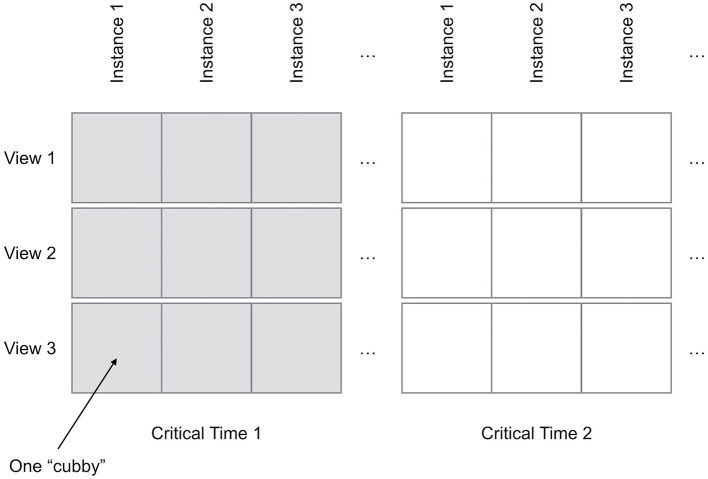
The Bento Box widget arranges multiple views of volume data within a grid of “cubbies”.

As diagrammed in Figure 2, each row and column have a specific meaning. Each row has unique *view settings*, which primarily define a specific sub-volume (i.e., a volume-of-interest) to crop from the original data along with the viewing direction. Additional parameters, such as the particular stress fields or other visualization properties to include in the view may also be set on a per-row basis. Each column presents data for a single *data instance* (i.e., the results of one simulation run) from the ensemble. If the user wishes to compare multiple timesteps (i.e., times-of-interest), this entire row-column structure can be duplicated multiple times.

Using this layout, the concept behind and intended use of Bento Box is for users to interactively create a comparative volume visualization where each column can be thought of as something like a volumetric, visual “feature vector.” The user's goal is first to select appropriate sub-volumes of interest within the data in order to build up a complete picture of the interesting variation in the data while, importantly, cropping out or deemphasizing regions that are less important. The data comparison task then becomes to compare these visual feature vectors. For each key feature (defined in a row), users make visual comparisons across the data instances in the ensemble (columns).

The key to creating a useful visual summary of the data is to explore and experiment: looking at the data from different angles, creating views of new sub-volumes, changing visualization parameters, navigating to different views of the widget, and adjusting the viewpoint used to render individual rows. We aim for users to naturally, through this exploratory and interactive process, arrive at a visualization that brings the most important sub-volumes to the forefront of the visual field while at the same time hiding or deemphasizing distracting or less scientifically relevant regions of the volume. Bento Box makes the *process* of exploring the data and creating the ensemble visualization fluid, natural, immediate, and iterative—users can perform the navigation and display management regularly and naturally during data analysis without even taking their gaze away from the dataset.

### 3.2. Zooming and Reframing the Widget

Viewing the entire Bento Box is like looking at an overview of the entire ensemble. Some major, high-level patterns are visible at this level, but the visualization within each individual “cubby” is too small to investigate in detail. This makes it important to be able to fluidly zoom in and out of the widget and otherwise reframe it on the display in order to see just the current portion of interest.

Since zooming and reframing is so critical to the user experience, Bento Box supports two complementary interaction techniques, each appropriate in a different context. The first is controlled by the input device held in the dominant hand (DH) and is appropriate to use when the Bento Box is displayed at a small scale (i.e., used as an overview). In this situation, a laser pointer is used as the virtual cursor for the DH and the user simply points this laser at the Bento Box to highlight a specific cubby to investigate in detail. A click and release of the DH's primary button triggers an animated transition that zooms the view into the selected cubby. Alternatively, by holding the primary button down and sweeping the laser across multiple cubbies, the user may paint a selection onto the widget, and the view will zoom to comfortably fit the bounds of this selection. In each case the BentoBox is scaled so that the front faces of the cubbies selected by the user fit within a 1 physical meter x 1 physical meter area centered in front of the user. To return to the default position and scale the user clicks and releases the same primary button while pointing the laser away from the widget. (When explaining the interface to users, we make this interaction easy to remember by telling them, “to go back, simply point the laser backwards over your shoulder”).

The one situation where this pointing interface is not efficient is when the view is zoomed in and the user wishes to make a small change (e.g., pan to the right by one or two cubbies). It is inefficient to do this by zooming all the way out and then back in to nearly the same position. Thus, the interface includes an ability to grab onto and translate the world directly. This is done with the primary button on the input device held in the non-dominant hand (NDH). To avoid unnecessary translations and rotations, this motion is constrained to only translate within the plane of the widget. Scaling is also possible. While the NDH primary button is depressed and the translation mode is active, pressing and holding the DH primary button activates the scaling mode. The scale of the Bento Box is then adjusted in proportion to the distance between the two hands. This second mode is like a constrained version of early VR object-manipulation interfaces (Mapes and Moshell, 1995; Cutler et al., 1997), which are also similar to modern 2D multi-touch interfaces (i.e., translate with 1 finger, scale with 2 fingers).

**Figure 3:**
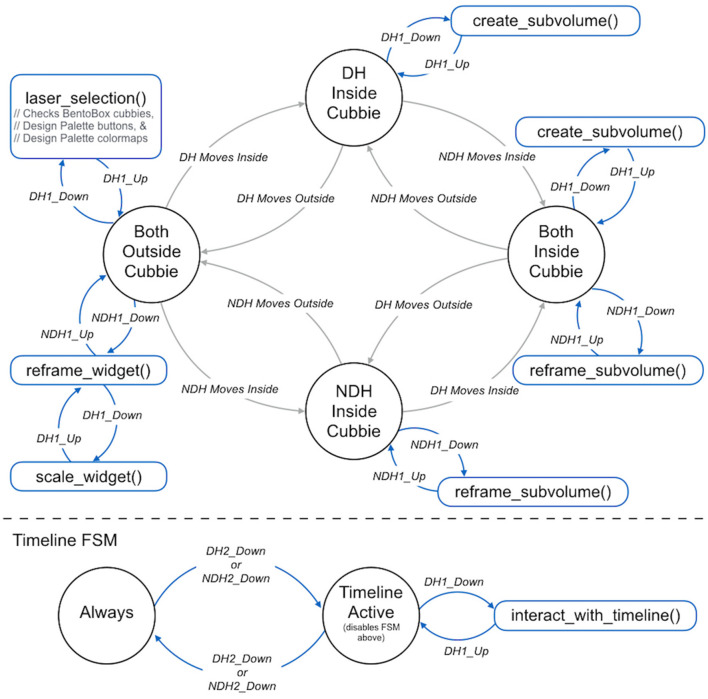
The 3D bimanual user interface is implemented as a finite state machine. There are four main states, and the system transitions between them based upon the positioning of the hands (DH, dominant hand; NDH, non-dominant hand) relative to the cubbies. As illustrated in the blue portions of the diagram, the actions triggered by the pressing the buttons on the VR wands held are different depending upon the context provided by the current state.

Note that the interface intentionally overloads the functionality of the DH primary button—it means different things in different contexts. This strategy has been used successfully in several other bimanual 3D user interfaces (Zeleznik et al., 2002; Jackson and Keefe, 2016). In general, the interface follows a pattern of using context to infer user intent whenever possible. This allows a complex interface to be specified using only two buttons on each hand-held input device and helps to overcome both learnability and “fumbling in the dark” problems that often arise in 3D user interfaces that use controllers with many buttons. It is important to consider the *state* of the system when designing and implementing this type of interface. Thus, Figure 3 presents a detailed Finite State Machine for the Bento Box interaction. The virtual cursors drawn in the scene change to provide visual feedback (e.g., from a laser pointer to a picking sphere) when moving from state to state.

### 3.3. Creating and Reframing Sub-volumes

As shown in Figure 3, a state transition is made when the DH cursor is moved within the bounds of the volume of a specific Bento Box cubby. Here, the concept is that the user is no longer in an overview mindset but rather in an inspection mindset. The cursor changes from a laser pointer to a small sphere to indicate this shift.

**Figure 4:**
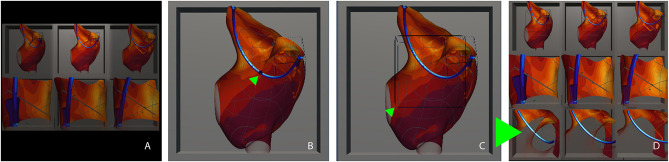
A new sub-volume of interest is created with an interactive selection. From left to right: **(A)** Assume the starting state is a Bento Box with two rows. **(B)** A click within any of the cubbies (in this case, one from the top row) using the primary button on the DH wand defines a center point for the selection (the black dot). **(C)** Dragging defines the size of the selection box to create. **(D)** After releasing the button, a new row of view settings is added to the bottom of the Bento Box.

The most important operation in this state is to indicate a new (sub)-volume of interest and thereby add a new row of view settings to the Bento Box widget. This is done via a click and drag operation. The 3D location of the cursor at the moment the DH's primary button is clicked is used as the center of the volume of interest, and the extent of the volume is set interactively as the cursor is moved away from this center. It is critical to display interactive visual feedback during this operation (Figure 4) so that the user may size the volume appropriately relative to features observed in the data visualization. The selection operation is completed by releasing the primary button.

Creating the new sub-volume adds a new row to the Bento Box widget. All of the view settings are copied from the originating row with the exception that the transformation matrix used to draw the data within the widget is adjusted to exactly map the sub-volume displayed in the new row to that selected in the originating row. The exact transformation is described when discussing rendering in section 3.6.

While the user is in this same inspection mindset, there is often also a need to view the data from a slightly different direction. Mirroring the use of the hands in the overview situation (DH = pointing/action, NDH = framing), this is accomplished with a grabbing operation controlled via the NDH. Again, the context provided by the positioning of the hand in space is used to distinguish a local grab that manipulates a sub-volume from the global operation that moves the entire Bento Box widget (see transitions in Figure 3).

### 3.4. Changing the Visualization With the Design Palette

**Figure 5:**
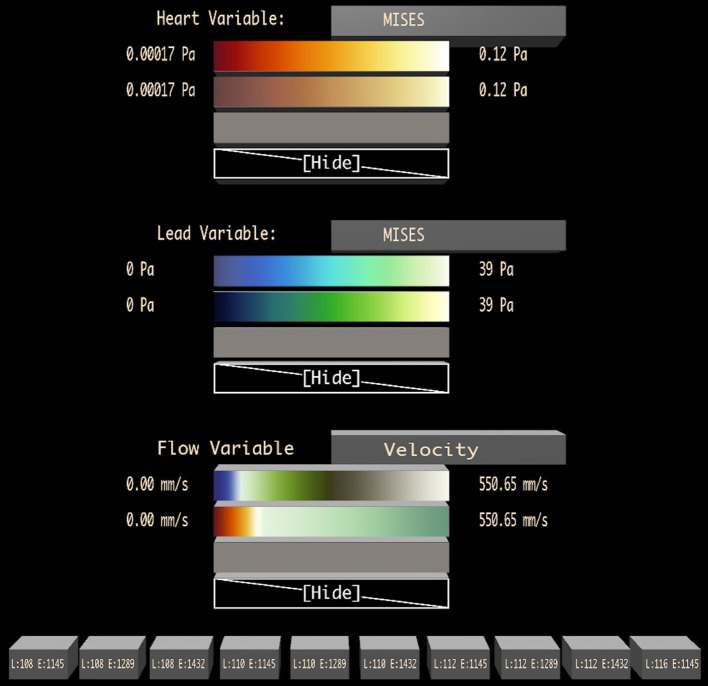
A visualization design palette is used to change the variable displayed on each major graphic element of the visualization (in this case: heart walls, lead, and flow) and to adjust the color map applied to the data. A set of possible color maps to apply to each variable is loaded during initialization, and the user may change the color map to display in each row of the Bento Box interactively by dragging one of these colormaps onto a specific row. Toggle buttons at the bottom of the palette control the visibility of specific data instances (the columns), labeled with instance parameters.

Figure 5 shows the visualization design palette, which is positioned to float in the air next to the user, coincident with the left wall of a Cave when running in a Cave display. The palette contains one section for designing the visuals used for each major graphical component of the visualization. Figure 5 shows an example from the cardiac application described in detail in 4. Here, there are three major graphical elements: the heart walls, the cardiac lead, and the flow path lines.

The heading for each section includes the name of the graphical element. Next to this is a button that can be selected with the virtual laser pointer attached to the dominant hand cursor and then pressed with a click on the primary button on the stylus. This cycles through a list of possible data fields that can be mapped onto the graphics (e.g., von Mises stress, Principle Stress, pressure).

Below this, the palette contains a set of color maps that can be used to present the data. With the laser pointer, a dragging operation is used to drag the colormap to a specific row of the Bento Box. While dragging, the laser cursor changes to include a colormap icon and rows of the Bento Box highlight as the laser passes over them to provide visual feedback. The use of dragging to control this operation is intentional, with one click and release, the user is able to specify both the specific color to apply (clicking while pointing at the design palette) and the row to apply it to (releasing while pointing at a Bento Box row). The last choice in each colormap list is a blank mapping (“Hide”), which removes the visual element from the scene completely.

Finally, an additional section at the bottom of the palette contains a set of toggle buttons, one per Bento Box column, to control the visibility of each data instance.

### 3.5. Using the Interactive Timeline

By default, each cubby in the Bento Box displays data from the same moment in time; however, additional time-points of interest (we call these *critical times*) can be added, extending the grid as diagrammed in Figure 2. Critical times are created and adjusted using an interactive timeline that is activated at a location in front of the Bento Box widget when the secondary button in either hand is clicked. The timeline acts as a modal widget, disabling the Primary Bento Box FSM shown above the dotted line in Figure 3 when the *Timeline Active* state is active. The timeline includes two virtual buttons that float in space and may be selected by the user's cursor. The first adds a new critical time to the display. This adds an indicator (color coded sphere) to the timeline at the correct time value and also adds the appropriate columns to the Bento Box. The critical times can be adjusted dynamically by grabbing onto the corresponding spheres on the timeline and moving them using the DH's primary button. While grabbing, the spheres can also be deleted by pulling them off the timeline by a distance of more than 0.3 meters and releasing. These interactions are demonstrated in the accompanying video from the Supplementary Material.

### 3.6. Rendering Multiple Clipped Volumes

Bento Box requires rendering multiple views of multiple volumetric data instances. Each row is rendered using different view settings, which consist of: (1) an affine transformation matrix that transforms the raw volume data to a particular view of a sub-volume of interest, and (2) visual settings, such as the set of color maps and variables to be displayed. Since the view may require the data volume to be drawn at a scale that eclipses the size of its cubby, the rendering must be clipped to fit within the cubby.

For each cubby in the widget, the transformation matrix that maps the raw data to widget space, *M*_*D*2*W*_, is composed of three parts and calculated as follows:

(1) $$\begin{array}{l} M_{D2W}=M_{V2W}*M_{C2V}*M_{D2C}. \end{array}$$

Here, *M*_*D*2*C*_ (Data-to-Cube), transforms the bounding box of the raw volume data to fit within a unit cube centered at the origin. This transformation is specific to the data provided with each new application; the same matrix is used for every cubby in the widget.

*M*_*C*2*V*_ (Cube-to-View) transforms from the unit cube space to the view of the sub-volume selected by the user—this includes scale, rotation, and translation); the same matrix is used for each row in the widget. When the program starts, with only the top row of the Bento Box visible, *M*_*C*2*V*_ is set to the identity matrix. When a new row is created, the matrix to use for this new row is computed using the matrix from the originating row. The following equation is used, where *S*() constructs a scaling matrix, *T*() constructs a translation matrix, and *p* and *r* define the center point and radius specified by the user (in View-Space coordinates).

(2) $$\begin{array}{l} M_{C2V_{newrow}}=S\left(1/r\right)*T\left(-p\right)*M_{C2V_{originalrow}} \end{array}$$

Finally, *M*_*V*2*W*_ (View-to-Widget) transforms from the view space defined for each row to widget space by translating by a vector of the form (*cubby*_width*row, cubby_height*col, 0) and scaling to match the current size of the widget. This is a simple translate-scale matrix, aligning the view to the specific cubby in which the data are to be rendered.

To clip each view to fit within its respective cubby, a clipping mesh is used. We chose a cube with rounded edges, but any convex shape of unit dimensions may be used. Before the view is drawn in a cubby, the back faces and front faces of the clipping mesh are rendered to two depth textures.

In our implementation, the graphics displayed for each data instance can be any 3D scene drawn using a traditional shader-based rasterization pipeline. There are two steps to adapt an existing rendering pipeline to work with Bento Box. First, the scene's model matrix *M*_*original*_ should be modified using *M*_*D*2*W*_ before the Model-View matrix is computed.

(3) $$\begin{array}{l} M_{new}=M_{D2W}*M_{original} \end{array}$$

Second, the fragment shader should be adapted to perform clipping to fit within two depth textures. For each fragment of the data scene, the fragment should be discarded if it falls nearer to the camera than a given Front depth texture, or discarded if it falls farther from the camera than a given Back depth texture.

## 4. Application and Results

The core Bento Box concept and technique described thus far, which we believe will generalize to other ensemble visualization problems, was inspired by the needs of a specific real-world data analysis problem, using an ensemble of fluid-structure-interaction (FSI) simulations to design improved medical devices. This section describes in detail the specific data management strategies developed for the application along with user feedback and quantitative performance measures from this first application of Bento Box.

### 4.1. Background: Cardiac Leads in the Right Atrium

**Figure 6:**
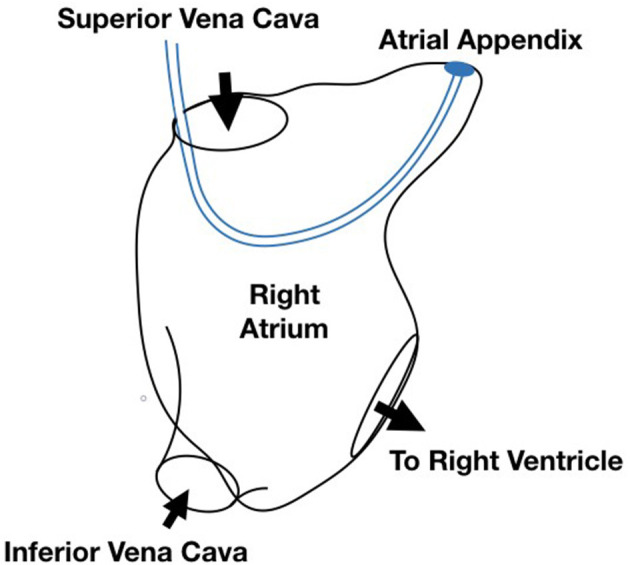
Diagram of the simulation scenario with a cardiac lead implanted in the right atrium of the heart.

Cardiac leads are the electrical cables that connect the heart to an artificial pacemaker device. The data visualized here come from a specific set of simulations designed to understand the impact of lead stiffness and lead length on the blood flow and stresses in the right atrium. The scenario is diagrammed in Figure 6. The goal of the study is to improve the underlying device technologies as well as procedures for implanting and extracting the devices. A 3 × 3 design was used for the initial study with three lead lengths (108, 110, and 112 mm) and three lead stiffnesses (8, 9, and 10N/mm corresponding to Young's Modules 1145.92, 1289.16, and 1432.39 MPa), resulting in 9 data instances. Later, one additional run (116 mm, 8 N/mm, and 1145 MPa) was added to the ensemble to understand the extreme case of extending the lead length as far as possible without touching the walls of the atrium.

The simulation extends Runesha et al. prior model (Runesha et al., 2016) and is built using the ABAQUS solver. The bounding geometry of the right atrium is a smoothed version of a real heart anatomy captured via CT scan, and the cardiac lead is modeled as a uniform wire entering the right atrium through the superior venae cavae and exiting through the tricuspid valve. Both the anatomy and the cardiac lead deform slightly over the course of a heartbeat. Each run consists of 800 timesteps for a 0.8 s heartbeat. Velocity and pressure within the volume along with stresses within the lead and along the walls of the atrium are saved at each timestep.

### 4.2. Sampling and Visualizing Solid Domain Data

As in most real-world ensemble visualization problems, some data management strategies are required in order to efficiently render many instances of the volumetric data. The approach described here is tailored to fluid-structure interaction (FSI) data and uses different strategies for solid domain data (Abaqus Implicit Solver) and fluid domain data (Abaqus CFD Solver). Since a VR rendering is required, the challenges of rendering large scale data cannot be solved with just an incremental loading or streaming approach, such as those used in recent 2D rendering contexts (Fisher et al., 2012; Glueck et al., 2014). Here, there is also a 3D computer graphics rendering problem where the data to be rendered at each frame are simply too large to fit within graphics card memory. The techniques described here are similar to those described previously in the literature for solid domain (Lee and El-Tawil, 2008; Beneš and Kruis, 2015; Liangyin et al., 2018) and fluid domain (Kuester et al., 2001; Sobel et al., 2004; Falk et al., 2016; Zhao et al., 2017) data visualization, but we believe this research provides the first example of extending and using both styles of visualization simultaneously to display *multiple instances* of FSI data in a head-tracked, stereo VR environment. Thus, we provide a detailed account as a case-study-level contribution.

The solid domain describes properties of deformable meshes like displacement and stress and defines the physical structure of the volumetric solid with a mesh. The element and node properties of the simulation are used to generate a triangulated mesh that can be rendered. Triangles are constructed from the supplied primitives and passed to the GPU. Each instance uses its own index and vertex array since the hearts deform as a result of the simulation.

Since the solid data for all instances, variables, and time steps are too large to fit onto the GPU, optimizing the GPU memory and update speed becomes a significant challenge. We solve this by minimizing both the memory footprint on the GPU and size of data streamed onto the GPU at any time. In addition, we use CPU memory caching strategies to avoid disk access as much as possible. This involves only loading the variables and time steps that are actively being displayed into GPU memory. For example, if a user is only looking at displacement and stress for three instances, only these data are dynamically loaded on the GPU. However, depending on the CPU memory size, many time steps may be loaded into CPU memory, allowing for quick update if a user chooses to animate the instances.

The solid domain data are loaded into GPU memory when the user changes the displayed variable, the number of visible instances, or the critical times. Only the selected variable's values are loaded for each visible instance at the valid time steps, keeping the memory load footprint as low as possible. Each solid domain variable (e.g., wall stress) is assigned its own GPU buffer with size equal to the number of critical times multiplied by the number of FEA nodes and then by the number of components (1 float for scalar values, 3 for vector values). Although for perceptual reasons, our default is to view multiple critical times in static juxtaposed views, the visualizations can be animated by simply swapping data every frame, and this can be done automatically or interactively using the timeline widget described earlier.

### 4.3. Sampling and Visualizing Fluid Domain Data

The raw fluid domain data are large, but these data are visualized using particles, so a significant data reduction can be achieved by converting the raw data into pathlines in a precomputation step. Our implementation uses an accelerated cell location technique, similar to a CellTree (Garth and Joy, 2010) data structure, to precompute path lines directly from the unstructured grid data using a Fourth Order Runga-Kutta integration method. A random seeding strategy is used; other seedings, such as targeted seeding in areas of high vortical structures, would also work.

The specific path lines to draw within each cubby are determined based upon the time and view. Since path lines inherently encode time; the display for a given critical time is determined by cropping each line to the portion that lies between the current critical time and a few moments before (in order to create a streaklet effect).

Bento Box requires rendering to be performed at multiple scales, and this raises an interesting challenge for drawing path lines. Recall that the path lines are just a visual representation for the underlying flow field, so when drawing them with a streaklet geometry, it makes sense to define the size of that geometry (its radius) in cubby-space units, not data units. Another way of thinking of this is that when zooming in, viewers do not wish to see a giant streaklet geometry, rather they want to see a more intricate visual rendering of the flow at that zoomed in scale. To accomplish this, both the size and density of the streaklets must be defined in cubby-space units.

Empirically, we determined that drawing about 2000 path lines per cubby provides the right balance for density—enough lines to provide detail to understand the flow and not so many that occlusion is a problem. This is a simple constant in our algorithm, and a different value may be easily incorporated to tune to technique for use with other datasest. What should not change from one application to another is the desire to maintain a constant visual density of path lines per cubby regardless of the scale of the data represented in that cubby.

To address this, the precomputed single set of randomly seeded path lines for each data instance is computed for the most-zoomed-in view expected. Then, any zoomed-out views that require fewer particles are drawn using just a subset of the precomputed paths; the size of the subset to draw increases as the view zooms into the data. The specific calculation is as follows. With the constant *N* as the application-specific desired visual density of paths per cubby, the number of particles to render, *n*, for a cubby displayed with scale factor, *s*, is

(4) $$n(s)=N{\left(\frac{s}{s_{external}}\right)}^{3}.$$

The final constant in the equation, *s*_*external*_, which is 4.0 for our data, is the scale at which the visualization transitions from an internal view of the flow data to an external one. With this formulation, *n* increases smoothly, randomly adding new path lines to the scene rendered and ultimately clipped into each cubby, as the view is zoomed in tighter.

The fluid domain rendering method makes it possible to visualize the flow at any scale and any critical time from the same pre-calculated data. Thus, changing the critical time does not use any additional GPU memory or require additional CPU-GPU memory updates. One path buffer array is stored on the GPU for each data instance in the display. The buffer is arranged according to a path index and each path has the same path length. The GPU also stores path value buffers for data variables such as velocity and pressure that may be used to color the particles.

The comet geometry is defined as an axis-aligned 3D mesh. Since each visualization will include thousands of these meshes, the mesh is rendered using instancing and deformed in a shader to fit within an appropriate start and end position along the path line. Our implementation uses a mesh with 72 triangles.

### 4.4. Insights and User Feedback

**Figure 7:**
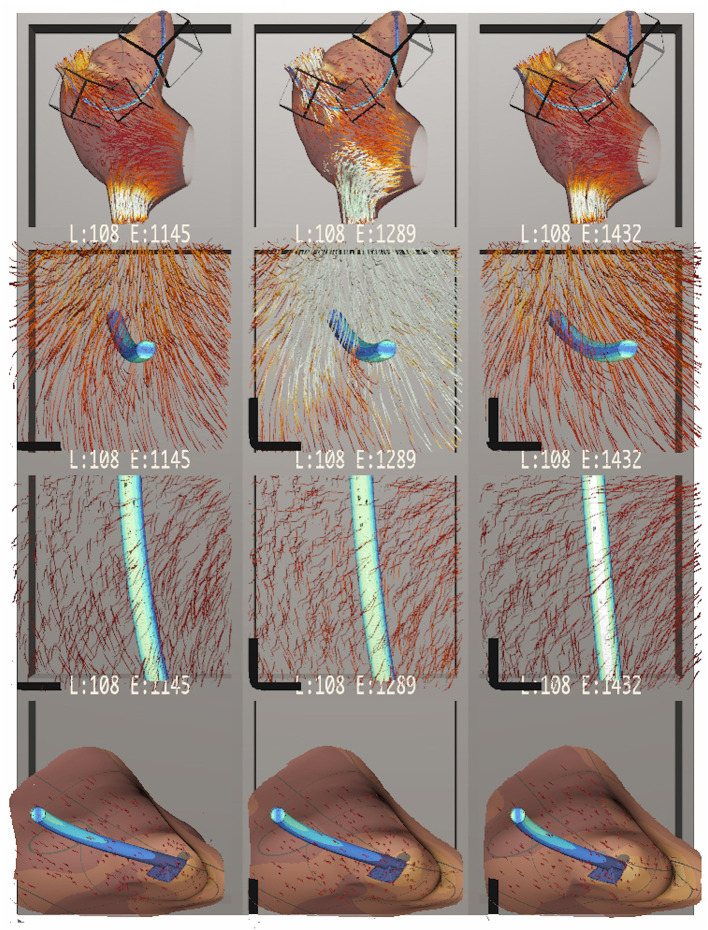
Bento Box arranged for a comparison of cardiac leads with three different stiffness parameters, increasing in stiffness from left to right. The top row shows an overview of the dataset. The middle two rows highlight stress on the lead itself, which appears to increase with stiffer leads. The bottom row zooms in on the attachment point of the lead in the atrial appendix, showing how in all cases the flow stagnates near the attachment point. Here, stress on the atrial walls also appears to increase with stiffer leads.

**Figure 8:**
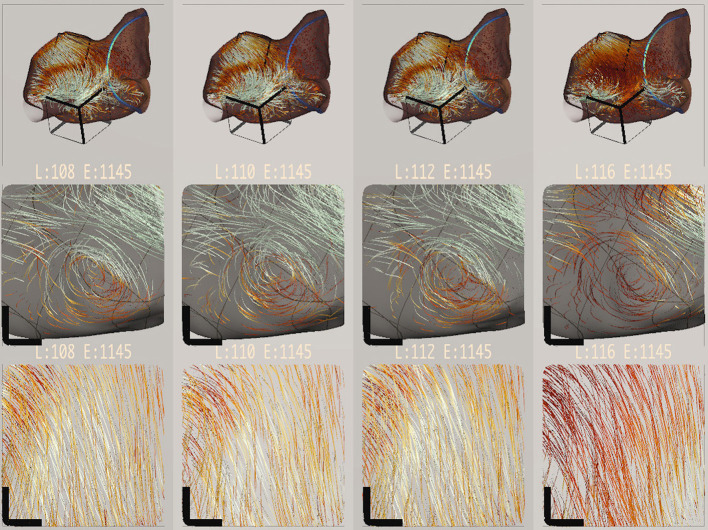
Bento Box arranged for a comparison of different length cardiac leads. Lead length increases from left to right. At this timestep in the simulation, the longest lead length creates a slower flow (i.e., darker red path lines).

Our interdisciplinary collaborators (also co-authors) have used Bento Box during several months of iterative development, most recently to analyze the scenarios highlighted in Figures 1, 7, 8. The team includes two mechanical engineering researchers and four computational scientists who also confer regularly with cardiac surgeons and with engineers in the medical device industry. Several new insights about the data were able to be made. These observations come from multiple working sessions in the Cave, which is used regularly for collaborative data analysis by small groups of users. As mentioned earlier, the application also runs on the HTC Vive, and this has been a useful platform for portable demonstrations at international conferences and for school groups, industry, and university alumni; however, users have preferred the Cave for data analysis because it facilitates collaborative discussion.

Initial observations confirmed the expected spatial positioning of the leads, which was easily judged in VR. The engineers commented that the “drapings” for the leads in all data instances were appropriate. Looking at cross-sections of the lead by arranging sub-volumes that cut through the lead like a slicing plane, the engineers also confirmed that the internal stress pattern has a neutral axis, an expected pattern for a bending scenario like this one.

Figure 7 shows a comparison of data instances with leads of increasing stiffness from left to right. The second row shows the neutral axis stress pattern mentioned earlier. The sub-volume was rotated with a NDH gesture so that the front of the cubbies act as cutting planes, slicing into the finite element data. The third row shows a top-down perspective of the lead. Here, white indicates high stress. Engineers found that the highest stress on the lead occurs when the lead is at its stiffest, confirming their expectation. Conversely, the leftmost situation should have the most displacement. This was difficult to verify because the displacements are all quite subtle, and a suggestion was made to include a “motion magnifier” feature in future work to exaggerate any movement. The fourth row shows the volume of the right atrial appendage near the attachment point of the lead. This is a region where flow circulation and stagnation can occur and fibrosis develops. Here, engineers noticed that the stress on the atrial wall near the attachment point is higher (more yellow and less dark red) with stiffer leads.

Figure 8 shows a complementary comparison. Here, the focus is on different lead lengths, which increase from left to right. Some interesting variation in blood flow within the volume is visible. Engineers noticed that the longest lead produced flow patterns that appeared slower (darker red) and slightly out of phase with the other simulations (visible when scrubbing through time). This slowing trend is visible in the overview in the top row, but it can be seen even more clearly in the next two rows, which focus on a vortex and an outflow. The wall stress is hidden in these rows, and a neutral background is used to make it easier to read the color-coded variation. During the most recent data analysis session, this visual insight prompted several minutes of follow-on discussion, and the computational scientists hypothesized that the longer stiff lead might stretch the atrium wall, making the atrium bigger, and that the increased volume creates a slower overall flow pattern.

In terms of usability, the layout and interface controls made sense to users, who learned the controls within a first working session. One suggestion for improving the interface design was made. Users were sometimes confused when translating a sub-volume relative to a parent volume. Recall, from the discussion in 3 that this is supported via a grabbing gesture with the NDH. Users understood the rotational aspect of this grabbing, but had trouble with the translational aspect. One user told us that when she was translating she looked not at the sub-volume where her hand was located but at different row where she could see the location of the sub-volume displayed as an icon. When the user focuses on the sub-volume's icon, this breaks the metaphor of using the hand to grab onto and “move the data” and instead puts the user in the mindset of grabbing and “moving the selection box.” Unfortunately, this does not work well in the interface because the translations will be the opposite of what is expected. The solution is not trivial. The widget is designed so that the boxes are arranged at fixed locations in space, so it breaks this design if the interface is switched to a mode of grabbing and moving the boxes. One option to explore in future work is to make the selection icons themselves objects that may be grabbed and moved with the hand. Then, if the user wishes to move the object, she simply finds a view where its icon is visible, grabs it and moves it. Alternatively, if she wishes to move the data, she grabs the data following the metaphor used in the current implementation.

### 4.5. Memory Usage and Rendering Performance

Characteristics for the ten data instances visualized are reported in Table 1. After processing the 39 GB of raw data, the amount of memory needed to accurately visualize the solid and fluid attributes is over 8 GB, exceeding a 4 GB GPU hardware limit on our 4-wall cave environment, a 2 processor Intel(R) Xeon(R) CPU E5–2640 @2.50GHz machine with two NVIDIA Quadro K5000 cards and 192 GB of RAM. Since this machine has more than 8 GB of RAM, it is possible to stream solid attribute data from memory into the GPU when needed. Streaming combined with the pathline sampling of the fluid, provides an extremely low memory footprint on the GPU, allowing us to visualize many instances and variables.

**Table 1:** Characteristics and memory usage for the ten data instances.

**ID**	**Raw**	**Solid (MB)**		**Fluid (MB)**	**Total (MB)**	
	**(MB)**	**Processed**	**GPU**	**Proc./GPU**	**Processed**	**GPU**
108–1145	5,252.6	892.6	7.0	115.2	1,007.8	122.2
108–1289	4,724.4	682.2	7.0	115.2	797.4	122.2
108–1432	4,911.5	682.2	7.0	115.2	797.4	122.2
110–1145	2,933.7	660.9	6.7	115.2	776.2	122.0
110–1289	2,933.7	660.9	6.7	115.2	776.2	122.0
110–1432	2,933.7	660.9	6.7	115.2	776.2	122.0
112–1145	4,926.1	687.5	7.0	115.2	802.7	122.3
112–1289	4,934.6	687.5	7.0	115.2	802.7	122.3
112–1432	4,934.6	687.5	7.0	115.2	802.7	122.3
116–1145	1,588.9	832.4	6.9	115.2	947.6	122.1
	40,073.6	7,134.7	69.1	1,152.3	8,287.0	1,221.4

**Figure 9:**
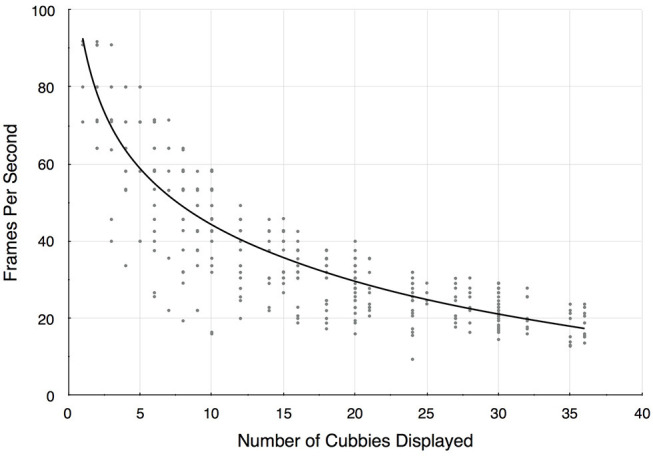
Rendering frame rates decrease as additional cubbies are added to the display. Since there are multiple ways to construct a Bento Box (e.g., there are 4 possible arrangements for 10 cubbies 10 × 1, 5 × 2, 2 × 5, 1 × 10), these results are for a systematic sampling of possible configurations. The trend line is a logarithmic fit (*R*^2^ = 0.76).

Using this application as a testbed, we also report some rendering performance measures, summarized in Figure 9. These timings were recorded on a 4 core processor Intel(R) CORE(TM) i7–7700HQ CPU @2.80GHz machine with 16 GB of RAM and a NVIDIA GeForce GTX 1070 graphics card, which was configured to drive an HTC Vive with a resolution of 2,160 × 1,200 pixels. The datasets are streamed into memory from a 128 GB M.2 PCIe SSD. The scatter plot in Figure 9 shows a systematic sampling of Bento Box configurations that are possible for this 10-instance data ensemble. All possible grid arrangements (10 × 1, 10 × 2, 10 × 3, 9 × 1, 9 × 2, 9 × 3, etc.) that result in a total of 40 cubbies or less were sampled. A cutoff of 40 was used since it is reasonable to assume that beyond this we reach a perceptual limitation in terms of what users can manage within the visual field. In fact, 20 cubbies is probably a better threshold. Since we found the rendering performance depends upon the zoom level, multiple samples at different scales were collected for grid arrangements that involved displaying sub-volumes. In general, rendering speed decreases as the view is zoomed in, since this requires rendering more path lines to achieve the same visual density as at zoomed out views.

The trend is above 30 frames-per-second for Bento Box arrangements of about 20 cubbies or less, and is in the 40–50 frames-per-second range for typical arrangements, such as for the results pictured in Figures 7, 8. In some arrangements sampled, the frame rates drop below what we would consider a bare minimum for VR environments (about 20 frames-per-second), but these cases are rare in practical use and have not detracted from analysis tasks using the system.

## 5. Discussion of Limitations and Future Work

There are two key limitations to the Bento Box technique that are worth reiterating. Although the concept, visual layout, user interface, and general rendering strategy can be applied to any 3D dataset, this only applies to cases where it is already possible to render the entire ensemble dataset. In fact, the ensemble needs to be able to be rendered multiple times per frame, in order to support multiple views at different scales. Our application demonstrates that this is possible to accomplish with a realistic, real-world, scientifically relevant ensemble, but in practice it takes some work and requires thinking carefully about how to sample and render the data. Some simpler datasets (e.g., 3D geometry only with no flow data) would perhaps work without any rendering optimizations, but many of the ensembles that scientists are interested in studying today will likely need to be optimized for fast rendering. One goal of reporting this case study is to provide guidance on how to approach this task, at least for fluid-structure interaction data.

Another limitation is that Bento Box is not the right technique for large ensembles. We intentionally describe the technique as designed for ensembles on the order of 10 instances for two reasons. First, rendering is even a bigger challenge for larger ensembles. Second, perceptually, it asks too much of users to try to interpret a juxtaposed visualization that goes beyond 20–30 “cubbies.”

This leads us to the most important direction for future work. We see great potential to combine Bento Box with a workflow that includes filtering. In this way, large ensembles might be able to be interactively filtered down to sets of 5–10 most interesting instances, then these could be explored in VR using Bento Box. This might be enabled, for example, by adding a linked scatter plot visualization of a dimensionally reduced view of a large ensemble from which the user could select individual or groups of instances to add to the Bento Box. Perhaps this could happen at a central control panel within a virtual room with multiple Bento Boxes created based on specific filters arranged around the virtual space. The workflow could also be extended in the other direction, making it possible to dive deeper into individual volumetric data instances to query specific data values with a probe or place other interactive visualization widgets directly within the detailed visualizations inside each cubby to access details on demand.

Finally, we now know, since Bento Box helped us to analyze the ensemble, that the variation within the particular cardiac lead data ensemble developed as part of the research project is quite subtle, and we are curious to learn how Bento Box would work in other situations, such as an ensemble where the variation between data instances is drastic. It would also be interesting to use Bento Box to explore abstract data, like a 3D field of data glyphs. In this case, the rendering optimizations described for FSI data would not be necessary, but, assuming the data are dense enough that zooming in is required to do detailed analysis of sub-regions, we hypothesize that the core technique would be just as valuable as it is for medical volume data.

In the future, we plan to conduct additional evaluations of the technique, for example, it might be possible to design a formal user study to assess speed and accuracy in a search and comparison task conducted with Bento Box vs. a standard juxtaposed or interchangeable (over time) comparative visualization. This would be a significant undertaking, likely requiring generating a synthetic volumetric ensemble dataset with features that can be interpreted by non-expert users. It may also be possible to implement a baseline VR or desktop-based visualization that uses an alternative visual approach to comparison, recruit additional experts with knowledge of cardiac medical device engineering and fluid analysis, and then design and execute a formal insight-based evaluation (Saraiya et al., 2005) of the support Bento Box provides for interpreting the data used in our case study. More generally, Bento Box is a good example of a visualization tool that, in practice, is often used in a collaborative data analysis mode; it would be interesting to more formally assess the strengths and weaknesses of various VR platforms (e.g., head-worn display vs. Cave) for this type of analysis.

## 6. Conclusion

VR environments are already effective for visualizing simulation data with complex spatial relationships, such as those presented in this paper, but only when visualizing a single data instance at a time. To make VR visualization useful for comparative visualization of a data ensemble, we conclude that new techniques for spatially arranging and cropping the data are necessary, since these help users focus attention on the most important 3D and 4D regions of comparison. Such an arrangement is only possible within VR with the aid of a tightly integrated 3D user interface. The Bento Box technique addresses both of these needs. Further, the application described here demonstrates that it is possible to use Bento Box to construct a visualization of a 10-instance, real-world, scientific data ensemble and provides early indication of the potential impact of visualizations in this style.

## Author Contributions

SJ developed the grid-based rendering framework and co-created the user interface with DK. DO created the GPU-based data sampling and rendering strategies advised by DK. HR and LM developed and ran the FSI simulations. BJ and AE defined and evaluated the medical device design scenario and parameterized model. FS iteratively designed the custom colormap set used to interactively highlight different aspects of the data.

### Conflict of Interest Statement

The authors declare that the research was conducted in the absence of any commercial or financial relationships that could be construed as a potential conflict of interest.
